# Twelfth Biennial Meeting of the Italian Medical Association

**Published:** 1887-11

**Authors:** A. Lagorio

**Affiliations:** Chiavari, Italy


### TWELFTH BIENNIAL MEETING OF THE ITALIAN MEDICAL ASSOCIATION.

The twelfth biennial meeting of the Italian Medical Association was inaugurated Monday, September 19th, in the Aula Magna of the University building at Pavia. His Majesty, the King, the Ministers of Public Instruction and of the Interior were respectively represented. Professor C. Golgi, President of the Executive Committee, welcomed the members present in a fitting manner. The mayor of Pavia, Professor Fedeli, Professor Bizzozero and others made addresses.

The meeting was divided into ten sections:

1. Medicine.
2. Surgery.
3. Hygiene.
4. Medical Jurisprudence, Neuro-pathology and Mental Diseases.
5. Obstetrics, Gynæcology and Diseases of Children.
6. Dermo-pathology, and Venereal Diseases.
7. Ophthalmology and Otology.
8. Anatomy, Physiology and Pathology.
9. Chemistry and Pharmacy.
10. Veterinary Medicine.

---

### SECTION ON MEDICINE.

Professor A. Riva was elected President; Professor Moragliano and Bozzolo, Honorary Presidents; Professor Rumino and Viziali, Vice-Presidents; and Doctors Balp and Cattanes, as Secretaries.

Professor Viziali read a paper "On Pneumonia," sent by Professor Tommasi, of Naples. The author believes that pneumonia is not always identical in nature and cause. He reminds us that he first condemned bleeding as injurious, and claimed that it frequently increased the mortality, and that in 1867 he introduced the therapeutical method of alcoholics with great benefit. They strengthen the cardiac systole.

Professor Golgi said that by his experiments he is led to believe that there is no such thing as malarial pneumonia, for he has never been able to find the characteristic alterations in the blood.

Professor Bozzolo believes that the primary pneumonia is due to only one cause, viz., the diplococcus of Franckel.

Professor Moragliano agrees with Bozzolo, and believes that many pneumonias, so-called malarial, are not so at all.

Professor Bozzolo spoke on "A New and Important Diagnostic Sign of Aneurisms of the Thoracic Aorta," inferred from the modifications of the negative pulse of the thorax in these cases.

Dr. C. Fossati read a paper on "Abdominal Oncology," and showed the "diagnostic difficulties" which may be encountered in some cases. In one of his cases there was a congenital pubic sac which communicated by four short ureters with the internal side of the left kidney, lowered and carried toward the middle; the patient died of peritonitis with extravasation of bloody urine from a rupture of the capsule. He exhibited the specimen.

Dr. C. Pavesi spoke "On the Passage of Antifebrin in the Urine." He concluded that the remedy in therapeutic doses does not pass unaltered in the urine.

Dr. A. Rovighi reported "Some Observations in the use of Strophanthus in Heart Diseases." He found it but little effective, although given in large doses (in some most grave cases), while digitalis and caffeine produced better effect.

Professor Bozzolo had had better results in his clinique; it is inferior to digitalis, but we must not forget that this is often not tolerated long by the heart and stomach.

Professor Bozzolo presented a girl affected with "myxœdema." He thought the case most interesting in having the affection localized at the head and the intelligence well preserved.

Professors Reymond, Rivi and Rummo discussed the subject and commented on the case.

Dr. G. Mya reported some clinical cases and researches, from which he concluded that the finding of urobilin in the vomited matters has a real importance in the diagnosis of the seat of intestinal occlusions.

Professor G. B. Gueirolo spoke on the "Genesis of the Vesicular Murmur," according to the results of some of his experiments made on a patient from whom the larynx had been extirpated. He concluded that the origin of the respiratory vesicular murmur must be below the two great bronchial tubes.

Dr. B. Silva, "On the Diuretic Action of Calomel." Many are the causes: Increased activity of the liver in the production of glycogen and urea; dilatation of renal vessels; irritation of the cells of the canaliculi by the direct action of the mercury, and also by that of the increased urea, insomuch that by continuing too long the experiments, in dogs, we can cause a glomerulo-nephritis; calomel, therefore, is contraindicated in nephritis.

Dr. Brugnatelli found the remedy efficacious as a diuretic in heart diseases, not in nephritis.

Professor E. Moragliano spoke "On the Disturbances of the Portal Circulation in Interstitial Hepatitis in Relation to Ascites and to the Mechanical Disturbance in the Field of the Systemic Circulation." Frequently in cirrhosis of the liver we find ascites in the last stages, although there be existing for some time an alteration of the intra-hepatic circulation; frequently, therefore, the dropsy is due to a disturbance of the general circulation, rather than the local.

Professor Bozzolo asked: "Can it not be due to a local alteration (chronic peritonitis)?"

Professor Moragliano never found such alterations in his cases.

Professor Silvestrini said that the acites could be easily explained by studying the peritoneal membrane, and especially the lymphatics of the peritoneum, which he always had found altered in his cases.

Professor Gueirolo presented a communication "On the Œdema of Patients Affected with Interstitial Hepatitis." The cause of external œdema in dropsy is the cardiac affection.

Dr. Trovati read a report of some of his "Experimental Researches on theAction of someRemedies on the Pulmonary Circulation." He concluded that ergotin, the extracts of hydrastis canadensis and of hamamelis virginica contract the vessels.

Professor Bozzolo presented a paper "On the Ætiology of Pneumonia." He reported fifteen cases of croupous pneumonia, in which he had only found the diplococcus of Franckel. He believed the question yet unsolved,

Professor Silvestrini mentioned three cases of pneumonia complicated with cerebro spinal meningitis; which he thought to be due to a diffusion of Franckel's diplococci through the nerves, rather than to a new infection.

Dr. Balestreri spoke at length on "The Rheumatic Origin of Pneumonia."

Dr. R. Massolongo, of Verona, spoke on the "Ætiology and Pathogenesis of Acute Broncho-Pneumonia," He maintained, as in 1855, that in general primary and secondary acute broncho-pneumonia is due to the same causes which generate the classic pneumonia: all the varieties depend upon the different conditions of the organism, and from the different virulence of the cause of pneumonia.

Professor Bozzolo, notwithstanding the results of Massolongo, persisted in believing that the primary pneumonia is due only to a morbific agent (Franckel diplococcus), and has no relation to the secondary pneumonia.

Massolongo believed that many cases of pneumonia, thought to be secondary, are primary, occurring in lungs much enfeebled by disease.

Professor Foa said that the possibility of a specific broncho-pneumonia is shown in the typhoid pneumonia, in which he found only the Eberth bacillus and never the Franckel diplococcus. He spoke at length on the pathological anatomy of broncho-pneumonia.

Dr. G. Rummo, of Naples, reported the result of his studies on the "Comparative Physiological action and Mechanism of Remedies acting chiefly on the Heart. Experimental researches." He used digitalis and digitaline, strophantus and storphantine, upas antiar, helleborine, erytrophieine, oleander, spartein, caffeine, adoninidine, convallariarine—strophantine, helleborine, digitaline, erytrophleine, upas antiar, and the oleander act on the myocardium; adonine and the covallararine act both on the myocardium and on the cardiac innervation; while spartein and especially çaffeine act above all on the cardiac innervation.

Professor A.DeGiovannin,of Padua,entertained the members on "The Diagnosis of Arteritis." It can be made from the beginning of the alteration, for this is sustained by the alteration of the vasovasorum; there are therefore vasomotor alterations, which must be distinguished from the reflex vasomotor alterations.

Dr. A. Boccolari presented "A New Writing Sphygmomanometer" which would eliminate the inconveniences of Basch's instrument.

Professor Bozzolo presented a paper on "Diabetic Albuminuria." The renal alterations are due to the acetonuria and diaceturia.

Dr. J. Masini spoke on "The Motor Cortical Center of the Larynx." On the cerebral cortex of the anterior hemispheres he found the center which presides over the movements of the glottis. The lesions of this center give symptoms of glottic paralysis.

Dr. C. Pavesi read a paper on "The Oscillations of the Weight of the Body in Pneumonia and in Typhoid Fever." When the temperature rises abnormally, the weight of the body does not diminish, for the water is retained; but when the temperature runs down the weight is less, for with apyrexia we have have a facility of elimination of the retained water.

These results are confirmed by Cantani.

Dr. O. Moretti, of Rome, read a paper "On Multiplex Paramyoclonus." He studied 19 cases. In two of these, and those were important, for in one (15 years) the muscles of the vegetative life were involved, and in the other (a man 55 years old), the co-ordinated movements only controlled the contractions in the inferior limbs. He showed the inhibitory power of the will in controlling the spasm.

Dr. Petrilli expressed his opinion on "Vaccination as a Curative Method during the Attacks of Small-Pox." It can also be done during an epidemic of small pox; it may abort the disease in individuals who have already shown prodromic symptoms.

Dr. G. C. Ampugnani, of Genoa, reported his "Experimental Researches on the Fever of Typhoid Patients and Pneumonia."

Professor Queirolo spoke on "Movable Dulness Sound of the Abdomen" in physiological conditions.

Professor Moragliano and Dr. Lussone presented a paper on "The Reflex Movements of the Vessels in Healthy and Febrile Individuals."

Professor A. Cantani, of Naples, spoke on "A New Form of Infective Pneumonia." It is characterized by broncho-pneumonia, preceded by a diffused bronchitis with remittent fever, with marked emaciation, and engorgement of the spleen. It is contagious, and is a primary affection of the bronchial tubes which spreads to the lower portion of the lungs, and to the trachea, larynx and pharynx; the blood must also be infected, for the spleen is much enlarged. It is probably due to a streptococcus.

Bozzolo and Cardarelli had both seen a case.

Professor A. Cardarelli spoke on "The Neuropathic Origin of Dupuytren Contraction." (Palmer.)

Professor A. DeGiovanni read the following papers: "Asphyxia of the Extremities," "Infantile Cirrhosis."

Professor E. DeRenzi on "The Ætiology of Pleurisy." He has experimentally reproduced pleurisy with the pneumonic virus. Primary pleurisy is not always tubercular.

The same author spoke on "The Treatment of Tuberculosis." The inhalations of sulphuretted hydrogen ($H_2 S$) make respiration eaisier and deep—creosote given in full doses lessens the secretions of the bronchial tubes of the cavities, and the cough, and improves the organism. Carbonic acid gas has almost no action. The alkaline treatment improves digestion and the general condition. Iodine and iodoform have given him the best results.

Dr. A. Dal Fabbro read a paper entitled "Is Exudative Pleuritis always Tubercular?"

Dr. Bertolero spoke on "The Treatment of Tuberculosis with Aniline." He has obtained good results. One must be careful to avoid effects of intoxication.

Professor A. Riva, of Pavia, said that he had lately tried the parenchymatous injection, with a suitable apparatus, of chloral-camphor, obtaining excellent results.

Professor Sorman made many experiments and found that terpinol, sulphuric ether, antipyrin, iodol, hydroxylamine, etc., have no effect. Chloral-camphor, tribromo-phenol are of much value in the treatment of tuberculosis.

Dr. Paoletti, of Perugia, spoke on "The Action of Alum in Tyyhoid Fever." He had given the remedy internally in sixty cases, and all recovered. Dose: ½ to 5 grammes daily.

Dr. Rainald presented a paper on "Hysteric Uræmia," with the history of a case.

Dr. F. Taji spoke on "Diphtheria and its Treatment with Resorcin." He obtained thirty-four cures in thirty-six cases. He used it locally as a spray.

Dr. F. Zinffré presented a paper entitled "The Epidemic Continued Fevers, Observed in Different Parts of Italy since 1872."

Dr. Magnani, read a paper on "Malarial Orchitis" cured with quinine.

Dr. Palese very greatly interested the society on "The Ictero-Hæmaturic Fever from Quinine Intoxication," mentioning many cases so affected by excessive doses of quinine.

Dr. Trombieri presented a paper on "The Treatment and Prophylaxis of Pertussis."

---

### SECTION ON SURGERY.

Professor E. Bottini, was unanimously elected President of the section; Professors Ceci and Ferrari, as Vice-Presidents; Professor Salomoni and Dr. D. Paolis, as secretaries.

Professor Bassini, of Padua, read a paper on "The Radical Cure of Inguinal Hernia." He described his new process, which consists in reconstructing physiologically the inguinal canal. He gave statistics of 72 operations, and concluded that the new operation is harmless, rapid and effective.

Professor Novaro, of Sienna, reported two cases as "A Contribution to the Surgery of the Stomach." The first patient was a girl 18 years old, upon whom he performed resection of the pylorus, for the relief of a cicatricial stenosis from an ulcer; she completely recovered in 29

days. The second case, also of cicatricial stenosis, was subjected to gastro-enterotomy; the patient died in two months from peritonitis, caused by an attempt to treat an intestinal fistula which had developed. He also reported his process of "Transplantation of the Ureters in the Rectum," successfully performed by him in the dog. The indications of this operation in man, are : 1st, malignant tumors in which resection is impossible ; 2d, uretero-abdominal fistulæ.

Professor Bassini read a paper on the "Osteo Plastic Resection of the Nose," for the removal of a returning nasal polypus, according to a new process of his, a modification of that of Langenbeck. The tumor is easily reached, and the bone is not detached from the soft parts of the nose and cheek.

Professor Petronis, of Naples, spoke on "The Pneumatic Medication of Wounds."

Dr. De Paoli, of Turin, presented a paper on "The Microbes of Altered Urine in the Genesis of Urinous Abscesses of Urethral Fever." He found micrococci (diplo and strepto cocci) and baccilli.

Professor Loreta, of Boulogne, sent a communication, relating a case of "Resection of the Left Lobe of the Liver, Infiltrated with Echinococci Cysts." He also related four operations of "radical cure of inguinal hernia, according to Bassini's method," with immediate good results.

Dr. Rota, of Turin, reported a case of a boy 5 years old, affected with chronic emphysema, relieved by performing "Estlander's Operation" modified with a thoraco-plastic process.

Dr. Carle, spoke on the same operation for the relief of empyæma.

Professor Tassi, of Rome, spoke on "Perineal Cystotomy," and Professor Roth, on "Suprapubic Cystotomy."

Professor Ferari, of Parma, read a paper on "Experimental Researches on the Wounds of Arteries, and on the Sounds Heard in Them." His results differ from those of Wahl and Dürms.

Dr. Petrilli presented a probe-bistoury for anal fistula which was favorably received.

Drs. Costa and Roth each reported a case of echinococcus of the liver successfully treated.

Dr. Paggi, of Boulogne, reported: I. "An exportation of the whole superior limb, together with the clavicle and scapula for the relief of a fibro-sarcoma," originating from the scapula, followed with good result, in a man 27 years old.—II. "A Disarticulation of the Femur," with an anterior flap, for the relief of a osteo-sarcoma of the femur, in a girl 17 years old, with recovery.—III. "A Resection of Knee Joint," according Durante's method.

Professor Tassi spoke on "Fungoid Synovitis."

Professor Ferrari communicated the results of 32 tracheotomies in cases of diphtheria, with 14 recoveries and 18 deaths.

Dr. Bergonzini spoke on "The Antiseptic Power of Iodoform," which he thought very feeble.

Dr. Falleroni, of Rome, spoke on "Osteoclasy" and showed Robin's apparatus.

Dr. Lampenonani, of Turin, made known his results on "The Surgery of the Hip Joint." He spoke at length on hip-joint disease, its smyptoms and pathology and gave his treatment in 13 cases.

Professor Ceci reported on "The Experimental Researches in Operations on the Bones."

Professor Tausini, of Lodi, presented a case of "Resection of the Stomach and Pylorus," followed with success. He presented the patient and the specimen. He spoke at length on the diagnosis and operative technique.

Dr. Guarmeri, of Lucca, presented the history of a case of "Circular Resection of the Intestine," with recovery.

Dr. De Paolis reported 12 cases of partial removal of the thyroid gland.

---

### SECTION ON HYGIENE AND HYDROLOGY.

Professor Sormani, President; Professor Morselli, Vice-President; and Dr. Campani, Secretary.

Dr. Nosotte read a paper on "The Transmission of Tuberculosis from Animals to Man." He concluded that tuberculosis is not transmitted by the digestive tract unless there be a solution of continuity.

Dr. Pratolongo spoke on "Vaccination."

Dr. Vinai read a paper on "The Hydrotherapy of Nervous Diseases."

Dr. Cozzolino reported his studies on the diffusion of diphtheria. Corrosive sublimate is the best disinfectant.

Dr. Morsalli read a paper on "The Examination of Drinking Waters."

Dr. Marchisio proved the efficacy of the thermal treatment in acute and sub-acute articular rheumatism, especially if associated with the salicylate of soda.

Dr. Burgonzio spoke on "The Technique of the Bath."

Dr. Ricchi found that arsenic was the best prophylactic of malaria.

Dr. Perroncito spoke on "The Biology of Thermal Waters." He found no micro-organisms in water coming from rocks, but did find them in water coming through earth.

Dr. Morselli presented a report of his studies on the hydro-electric bath. It benefits by the psychical impression which it produces.

Dr. Bordoni-Uffreduzzi found micro-organisms of all kinds in ice.

Many other papers were presented in regard to the therapeutical indication of the different mineral and thermal waters of the state. All were freely discussed.

### SECTION ON MEDICAL JURISPRUDENCE, NEUROPATHOLOGY AND PSYCHIATRY.

Dr. A. Raggi, President; Dr. F. Vizioli, Vice-President.

Professor F. Vizioli read a paper on "The Facial Trophoneurosis." Fifty cases are so far on record. He had two cases, and believed it to be due to a trophic alteration of bulbar origin. He does not believe that the disease is caused by any lesion of Gasser's ganglion, as now believed.

Dr. Frigerio had a contribution on "The Seat of the Sense of Smell."

Professor A. Verga sent a paper on "Acrophobia," which means an aversion to height.

Dr. Marro read a paper on "The Alimentation of Insane Patients."

Dr. Raimondi related two cases of traumatic rupture of the heart.

Dr. Algeri mentioned a case of epilepsy of traumatic cause in an insane patient, treated with success by trepanning the skull.

Dr. Gasparini had a paper on "The Pathogenesis of Acute and Chronic Facial Neuralgia, and on the Method of Treatment with Galvanism."

Dr. Raimondi spoke on the biology and toxic action of the muriate of lupinine.

Dr. Raggi read a paper on his "New Studies on Hypnotism," in which he advocated strongly the theory of polarization.

Dr. Stefanini presented two patients cured by the hypnotic suggestion. The first was a girl 14 years old, affected with hystero-epileptic convulsions, with contractions of the right upper and lower limbs—hemianesthesia, blindness and deafness of the corresponding side. In three months the patient fully recovered with the hypnotic suggestion. The second was a woman 32 years old, hystero-epileptic. Her recovery was also completed by using the hypnotic suggestion. Both patients were hypnotized before the members of the section.

Dr. R. Rainaldi presented a hystero-epileptic patient in a state of absolute lethargy. This patient was subjected to some very interesting experiments. The state of ecstasy and catalepsy were beautifully shown.

Dr. Bianchi had a paper on "The Nature of Pulmonary Lesions in Paralytic Patients." Two-thirds of these patients die of pneumonia. In 11 cases he found, besides the pulmonary lesions, degeneration of the pneumogastric nerves at their peripherical branches.

Dr. G. Rezzonico read a paper on "Observations of Pathological Anatomy in the Progressive Paralysis of the Insane."

Professor Tambroni mentioned a case of paralytic dementia in a patient affected with progressive paralysis.

Dr. Sighicelli spoke on the treatment of epilepsy with the galvanic current.

Dr. Bergonzini presented an interesting collection of skulls of criminal patients.

### SECTION ON OBSTETRICS, GYNÆCOLOGY AND DISEASES OF CHILDREN.

Professor Cuzzi, President; Dr. De Cristoforis and Professor Macari, Vice-Presidents; Dr. Bossi, Secretary.

Professor Macari read a paper on "The Fœtal Head," demonstrating the importance of the study of this element in the mechanism of labor.

He also spoke on "Obstetrical Craziness," showing how many obstetricians at times make great mistakes by getting too much excited by the slight difficulties which the clinical case may sometimes show.

Professor Paladisso sent a paper on "The Destruction and Renewal of the Ovarian Parenchyma in the Mammiferous."

Dr. F. Candia, of Naples, reported a case of supravaginal amputation of the uterus for the relief of a myoma, performed according to Schrœder's method.

Dr. H. Pinzoni spoke on "The Influence of Ergot on the Phenomena of the Puerperium." He concluded that ergot given during a physiological puerperium has no effect on the temperature; the frequency of the pulse is always increased, the urine is increased, due to a greater arterial blood pressure. Ergot has no effect on uterine involution, an opinion quite different from what we had always believed. The uterine after-pains become less. The milk secretion is impeded, being probably due to contractions of the vessels and to a less flow of blood to the gland; lastly, the influence of the remedy in puerperium is good, by preventing the retention of clots and their decomposition.

Professor Tibone always obtained good results by administering ergot in uterine involution.

Dr. Colucci, of Naples, read "A contribution to the Physio-pathology of Abortion." He found, in four cases, atrophy of the placental villi, which, by obliterating the blood vessels, diminishes the field of nutrition of the fœtus, and in consequence causes an abortion from the 10th to the 15th week.

Dr. Bordi presented a communication "On the Use of Jequirity in the Treatment of Chronic Metritis." From the beneficial effects of this remedy in ocular diseases, he thought to try it in chronic forms of metritis. He had used sterilized solutions which keep their activity, and made the injections with Braun's syringe. In the first 5 hours no reaction followed; then commencement of an acute metritis with fever (the highest 38.7° centigrade), with apyrexia on the third day. As local effects, he noticed first a fibrinous exudate which covered the os uteri; when this became eliminated the cure was complete. The author thought this remedy indicated in metritis granulosa in its second stage; the effect is not so good in the fungoid endometritis; is contraindicated when there is existing parametritis.

Drs. Clivio and Monti presented "A Contribution on the Ætiology of Puerperal Peritonitis." They found the disease was due to numerous and well known pyogenous micro-organisms, (streptococci) which is of great importance in obstetrical practice.

Professor Chiarleoni presented a communication "On Hysterism." He gave the history of a patient who, by a sudden fright, became affected with an hysterio-epilepsy, which grew worse in its convulsive and paralytic manifestations, and greatly endangered the life of the woman. He excluded any material lesion of the genital apparatus, and thinking of the many cases of hysteria cured by suggestion, he concluded that probably many of these patients who had undergone the operation of castration had sometimes recovered simply from the influence produced on the imagination of the woman. To remove doubt, Professor Chiarleoni proposed and performed a simulated operation; he fully chloroformed the patient, and then made only a slight incision of the abdominal walls. The result was complete; the patient left the clinic fully recovered; menstruation appeared again regularly, as it had ceased since the beginning of the sickness. Probably the amenorrhœa was due to a disturbed innervation of the internal genital organs; castration, therefore, could not have been proper in this case. By this cure, we may bring some doubts on the legitimacy of castration in nervous forms in general.

Professor Cuzzi is not yet convinced of the great advantages claimed for castration in the treatment of fibroids.

Dr. Pinzani spoke of "A Case of Incarceration of a Retroverted Uterus, at the Fourth Month of Pregnancy." The woman was a primapara. He thought the retroversion preceded pregnancy. All attempts at reduction having failed, and the patient being greatly exhausted and near to death, he punctured the uterus through the posterior fornix. Abortion followed, and the retroversion changed into retroflexion. The patient died from shock during puerperium. At the autopsy there was found: the bladder with thickened walls but with a normal mucous membrane, and the left kidney affected with nephritis. Drs. Calderini, Pasquali and Cuzzi discussed the case; and Dr. De Cristoforis reported a case of habitual abortion due to a retroflexion. By applying a pessary, the patient was brought to full term. The displacement was finally cured.

Dr. Accouci presented a new modification of Braun's cranioclast.

Professor Tibone presented "A Contribution to the Study of the Causes of Death of the Fœtus During Labor." He mentioned a cause little known, viz,: Deficient ossification of the cranial bones of the fœtus; hence compression transmitted to the brain, and also to the bulb through the cephalo-rachidian liquid. He does not advise version, but the preparation of the genital tract with the colpeurynter, and the cautious use of forceps.

Dr. Borde read a paper on "Endo-uterine Tamponing in Post-partum Hæmorrhages." In two cases he resorted to a tampon soaked in a solution of perchloride of iron; the hæmorrhage ceased and the puerperium was normal. It gives better results and is less dangerous than injections.

Dr. Candia said that an injection of water at a temperature of 50° centigr. was prompt in its action. He was also corroborated by Dr. De-Cristoforis.

Dr. Colucci read a paper on "The Glands of the Fallopian Tubes." He made several comparative studies on the oviduct of women, cats, rabbits and female bears; and found that in some animals the mucous membrane penetrating in the underlying layers gives rise to true glandular formations. (Muciparous glands.)

Dr. Pestalozza described his "New Operative Process in the Treatment of Cervicitis and of Parenchymatous Metritis." He does not approve of the use of caustics, and demonstrated the necessity of diligent ablation with the bistoury.

Dr. Candia spoke on his "Operative Radical

Cure of the Prolapse of Uterus and Vagina." He slightly modifies Martin's method.

Dr. Cuzzi usually used Hegar's method; he prefers Neugebauer's operation in women who have passed the menopause.

Professor Pasquali reported "A case of dermoid cyst treated with the caustic and with drainage."

He reported also two cases of chronic inversion of the uterus; the first, being irreducible, he removed the inverted portion; the patient died of septic peritonitis and re-inversion of the stump. The second case was treated with the colpeurynter, and it was reduced almost spontaneously on the 25th day. He attributed the inversion of the stump as well as the reduction with the colpeurynter to the action of the large ligaments."

Professor Inverardi read a paper on "The Study of the Mechanism of Labor."

Professor DeCristoforis gave a report of the "Night and day obstetrical guard of Milan." Gave its history and the good results obtained. In seven months there were made 1,219 visits at the homes of patients. He hoped that similar institutions may rise in every city for the benefit of the poor.

Dr. Cioja spoke on "Obstetrical Prophylaxis." On the basis of recent theories on puerperal fever, he showed the necessity of considering as infectious, and equal to other epidemic and contagious diseases especially in regard to sanitary laws and regulations.

In consideration of the importance of the subject, it was voted that it be a theme of general discussion at the next meeting.

Dr. Piazza read a paper on "The Convenience of the Diagnostic Puncture of Ovarian Cysts." The puncture may cause an intra-peritoneal spreading of the fluid ; he also said that, as in the splenic pulp, there may happen reproduction by dissemination. He spoke of other dangers, and recommended to limit or to abandon the puncture of ovarian cysts, which is not indispensable for diagnosis.

Dr. Carborelli, of Messina, spoke on "The Cyphotico-rachitic Pelvis." He mentioned a case where cyphosis existed at the inferior strait, and rachitis at the superior.

Dr. E. Casati, of Rome, spoke of his "Modification of Alexander's operation," which consists of a transverse incision from one inguinal ring to the other. He sutures both ligaments to the pillars of the external inguinal ring, and lastly crosses on the pubes both segments previously shortened and sutures them on their external passage with the subcutaneous connective tissue, which prevents the reproduction of the vitiated position. He performed this operation on a patient with a good result.

Dr. A. Guzzoni, of Pavia, explained his "New sign for the diagnosis of gemellar pregnancy." It consists in the limited fluctuation to one half of the uterus, after the rupture of one sac and the escape of its waters. He found it in three cases. He also noted an earlier disappearance and dilatation of the cervical canal in the last days of pregnancy.

Dr. M. DeCristoforis read a paper on "Massage of the Female Genital Organs."

Dr. L. M. Rossi read a paper on "Endo-uterine Injections." He does not fear the passage of liquids into the peritoneal cavity. He avoids uterine colic by using, before the injection, a solution of cocaine.

He also presented "two new forms of pessaries for the treatment of lateral displacements of the uterus." They are a modification of Hodge's pessary.

Dr. Bancolini reported "A case of osteomalacia in the province of Pavia." The case was very interesting, and especially because this province was always considered free from this affection.

He also spoke of two cases of threatened abortion from the administration of quinine.

Dr. Candia spoke on "Scooping of the uterine cavity in chronic hæmorrhagic endometritis." He demonstrated its efficacy and reported six cases so treated by Martin's method; narcosis is not indispensable; he found good effects in the use of a 4% solution of cocaine.

Dr. Sangregorio read a paper on "Small-Pox and Pregnancy." In the years 1884–85–86 and in the first six months of 1887 he had had under his charge 72 cases of small pox during pregnancy. In 31 the pregnancy was interrupted and 26 women died. In these 72 cases seven were cases of varioloid, and all recovered; only one had her pregnancy interrupted; 40 were cases of moderate small-pox, three died and 10 had their pregnancy interrupted; 22 had confluent small-pox, 20 died and 17 had pregnancy interrupted before death; three had hæmorrhagic small-pox, and all died after aborting.

---

### SECTION OF DERMATOLOGY AND VENEREAL DISEASES.

Professor C. Manassei, President; Professors A. Scarenzio and G. Casarini, Vice-Presidents; Drs. Ciarrocchi and Mibelli, Secretaries.

Dr. R. Campana, of Genoa, read the first paper on "The Tuberculosis of the Skin and of the Mucous Membrane of the External Genital·

Organs." He mentioned three cases: one of verrucous tuberculosis of the hands, in which he found Koch's bacilli; another of ulceration of the genital organs, which he diagnosed as tubercular. He spoke of its importance, because of the possibility of contagion through venereal contact. He made inoculations in calves with material taken from the ulceration, and in nine days the animals died, and he found tubercular spots and partial peritonitis.

Prof. De Amicis, of Naples, reported four cases of fungoid mycosis of Alibert. He made a bacteriological examination with a special method of his own (gentian violet 3 c.c., aniline oil 3 c.c., alcohol 99 c.c., distilled water 80 gramms) and found a special micrococcus.

Dr. Scarenzio showed a patient affected with gummous syphilis of the nasal bones and palate, with perforation, treated successfully by a plastic operation.

Dr. D. Majocchi, of Parma, read a paper on "Dermo-actinomycosis." Described the form of the actinomyces, and the results of his studies on the spores of this fungus, on their extreme smallness and their resistance to the pressure of covering glasses and reagents. He said that the alcoholic solution of fuchsin is the best coloring agent of the fungus, and especially if it is undergoing a calcarious degeneration. He spoke of two cases of human actinomycosis observed by himself: the first had an anthracoid aspect with a rapid course; the second presented itself as fungous, ulcerous, similar to those forms once called scrofulous. He thought that the parasite enters the system by the mouth.

Other papers were presented and read, as follows:

"Marginal Tricophyton Tonsurans," by Prof. R. Campana.

"Pathology, pathogenesis, morphology and treatment of Lichen," by C. Manassa.

"Pathogenesis of Alopecia areata," by Prof. G. Behrend of Berlin.

"Salol and iodoform in the local treatment of venereal and cutaneous diseases," by D. Barduzzi. He also read a paper on "Late Hereditary Syphilis."

"Universal exfoliative dermatitis from psoriasis;" "Pustulous eruptions of iodic origin;" "A case of universal lichen ruber," by Prof. T. DeAmicis.

"Herpes tonsurans maculosus et squamosus," by G. Peroni, of Turin.

"An ointment urethral injector in the treatment of chronic gonorrhœa;" "Treatment of Gonorrhœa," by P. Tommasoli, of Pesaro.

"Resorcin and epithelioma of the skin;" "On the abortion of syphilis by the cauterization of the infecting ulcer with the thermo-cautery of Paquelin," by L. Mannino, of Palermo.

---

SECTION ON OPHTHALMOLOGY AND OTOLOGY.

Dr. N. Manfredi, President; C. Reymond, Vice-President; Guerenzli, secretary.

The following papers were read:

"Some Experiments on the Mydriatic Movements of the Pupil," by Dr. Verdese.

"On the Antiseptic Power of Chloro-mercuriate of Cocaine," by Professor Tartuferi.

"The Histogenesis of the Retina and Optic Nerve," by Dr. F. Falchi, of Turin.

"On the Artificial Maturation of Cataract," by Dr. Denti.

"The Small Superior Flap modified in the operation of the Lenticular Cataract;" "The Disinfectants in the Conjunctival Granulations;" "Studies on the Micrococcus of Granulations," by Dr. Petrilli.

"The Hygiene of Sight," by U. Petrini.

"The Genesis of Myopia," by Dr. Ferri, of Novara.

Professor Cozzolino spoke at length on "The Intratympanic Surgery."

---

SECTION ON LARYNGOLOGY.

F. Massei, President, G. Loughi, Vice-President; I. Masini and V. Cozzolino, Secretaries.

Dr. Paladino reported a case of a stone lodged in the right bronchus, and extracted with a special instrument invented by himself. He showed the instrument (a bivalve speculum cannula) and was much praised for it.

Dr. E. Kruck read a paper on "The Adenoid Vegetations of the Naso-pharyngeal Cavity." He showed the instruments for the the operation, and anatomical and microscopical specimens.

Professor Cozzolino reported several cases of oral psoriasis. He insisted that it is independent of syphilis, and that the use of the galvano-cautery is the best treatment.

Dr. F. Massei spoke on "The Erysipelas of the Larynx."

Dr. G. Massini spoke on the treatment of hyperplastic laryngitis with scooping, galvano-cautery and lactic acid. He favored the first.

Professor O. Massini reported a case of laryngeal cyst.

Dr. Ferreri spoke on the treatment of laryngeal polypi, with special scoops used in the clinique at Rome.

Dr. Massini reported "A case of hereditary syphilis of the skin, tongue, face, nose and ears with tracheal stenosis, cured."

All these papers were fully discussed by the members present.

### SECTION ON ANTOMY, PHYSIOLOGY AND PATHOLOGY.

G. Loja, President; P. Foa and G. Romiti, Vice-Presidents; Drs. Fusari and Monti, Secretaries.

Professor P. Foa, of Turin, spoke firston "Putrid Infection," mentioning his two clinical cases in which he found a bacillus which by its character was recognized as Hauser's *proteus vulgaris*. He reported several experiments made with it.

Professor G. Bizzozero reported his studies made with Dr. Vassale, "On the Tissues of Secreting Glands." They concluded that most glands of the adult body possess elements of great stability. The salivary and mammary glands also follow this general law; but during pregnancy numerous are the elements in the way of scission in the mammary glands, due to its preparation for the period of functional activity.

A. Marcacci read a paper on "The Action of Alkaloids on the Vegetable and Animal Kingdom." He found by experiments that quinine and cinchonanine are potent anti-fermentatives; that strychnine and morphine are almost indifferent, and that atropine and veratrine seem to excite some fermentations. A similar action is shown by these alkaloids on germination, on the development of roots and bulbs. The action of poisons is much different from that commonly believed.

G. B. Grassi spoke "On the Parasites of Man." He said that the amœba coli is not found exclusively in dysenteric patients, but also in individuals whose intestinal functions are normal. He doubted the living nature of Pfeiffert's monocistis zosteri and the plasmodium malariæ of Marchiafava and Celli.

L. Tenchini, of Parma, reported "a case of congenital bilateral renal ectopia in an adult woman," and presented the specimens. He found both kidneys in the pelvis. The patient was well formed and never had any disturbance during life, but could never bring pregnancy to full term. The kidneys resting on the small pelvis impeded the distention of the gravid uterus.

P. M. Giuri also reported "a case of ectopia of the left kidney."

Drs. Martinotti and Sperino reported their histological researches on a diprosopus tetrophtalmus pertaining to the human species which lived a few hours. It had two oral openings, two noses, four eyes, four cerebral hemispheres, a cerebellum, one pous and a spinal column. They thought probably due to a fusion of two embyros.

F. Tartuferi spoke on "The Minute Structure of the Retina."

A. Bonome spoke on "The Ætiology of Tetanus." He had studied many cases of tetanus, and reported many experiments which he had made. He thought that the cause was the spindle form bacilli, found in large numbers.

Professor Herzen, of Geneva, spoke on "The Exhaustion of Nerves." His experiments differ from those of Vedenski and Bowditch. He concluded that the exhausted part is not the muscle, but the nerve.

G. Fano, of Genoa, spoke on "The Electric Rhythm of the Heart."

L. Vincenzi reported several experiments with cholera.

Dr. Oddi, of Peruzia, read a paper on "Cholecysto-gastric Fistula." He performed it in eight dogs. None had the digestion disturbed. The examination of the gastric contents made during different periods of digestion always showed the presence of bile, acid reaction and a normal quantity of peptones.

These results were confirmed by Herzen.

Dr. Oddi also reported another important experiment. He removed the gall bladders from dogs, and a little time after he noticed that the hepatic, cystic and choledocus ducts were much dilated. He discovered that at the opening of the choledocus in the intestine there existed a true sphincter.

Dr. Tizzoni reported his experiments on "The physio-pathology of the suprarenal capsules." By extirpating the suprarenal capsules in rabbits he found an increased pigmentation of the lips and tongue.

E. Marchiafava, of Rome, spoke on "Malaria."

Professor P. Foa, spoke on "The Structure of the Red Blood Globules." Having used several methods of straining, and having dried the blood at different degrees of heat, he detected a reticulum, the net-work of which was attached toward the center to a little ring encircling a space containing a little body which he considered as a residuum of the nucleus. Lastly, an hyaloid layer at the periphery; under this a layer of hæmoglobin, then a reticular layer, and lastly the protoplasm.

Drs. Foa and Bordoni Uffreduzzi reported their studies on "The Ætiology of Cerebro-Spinal Meningitis." According to them, the meningococcus is the same which Steinberg cultivated from saliva, and Franckel as diplococcus pneumoniæ. It is a constant cause of cerebro-spinal meningitis, while it is not the only cause of pneumonia.

Dr. G. Mingazzini, of Rome, spoke on "Anatom ical Observations on Brains of Criminals." He concluded that we cannot yet establish a special type of brains of delinquents.

Dr. Bonome spoke on "Pulmonary Lepra." Although this affection has been denied by recent writers, the speaker found it in a patient affected with chronic broncho-pneumonia. By using Ehrlich's and Unna's methods he found the Hausen's bacilli.

Professor Foa confirmed Dr. Bonome's observation, and Dr. Bordoni Uffreduzzi spoke at length on these bacilli.

Dr. A. Di Vestea spoke on "The Transmission of Hydrophobia through the Nerves." By inoculating rabbits according to Pasteur's method, he found that the medulla oblongata became virulent before the spinal chord. In the first stage he found that the rabbits had a characteristic elevation of temperature, and with the lowering of the temperature there was a paralysis of the posterior brain. By inoculating the sciatic nerve the lumbar medulla became virulent first. He related many other experiments tending to show the nervous theory of rabies.

Dr. V. Gianturio, followed next on "Histological Researches on Hydrophobia." He reported an autopsy in which he found an infiltration of the ependyma and of the perivascular lymphatic spaces of the nervous centers. He thought, therefore, that hydrophobia has characteristic lesions in the vessels. The nervous alteration would be secondary and only degenerative.

Dr. C. Bareggi, of Milan, read an elaborate paper on "The Bacteriological Examination of the Blood as a Rational Basis of Pasteur's Treatment." He claimed to have discovered in the blood of bitten patients a special micro-organism, which enabled him to diagnosticate the amount of infection of each patient.

Dr. T. Zässlein spoke on "Bacteriological Researches in Cholera."

Miss Dr. G. Cattani read a paper on "The Influence of Cold on the Spirocetes of Cholera." She studied the resistance of cultures buried under snow, and found that the komma-bacilli lived, but modified slightly their biological properties.

Several other papers were read and discussed.

For brevity's sake I shall not report the transactions in the section of Pharmacy and Chemistry and of Veterinary Medicine. Some papers read in the latter section were of unusual interest.

LECTURES.

To make the meeting of the association more interesting, several lectures were delivered before all the members in the large amphitheatre of the university. As I desire not to use too much space in your valuable journal, I shall only attempt to give a brief summary of two or three of them, which were delivered by the most eminent clinicians and specialists of Italy.

In the afternoon of September 19, Professor Henry Morselli, of Turin, a great authority on psychology, delivered his lecture on "The Mental Pathology, its Present and Future Status."

The aim of the lecturer was to defend psychiatry from the charge of some physicians, that it occupied itself with abstracted studies, by studying particularly the psychical phenomena, and neglecting the positive study of the brain and its alterations in insanity. Professor Morselli demonstrated that the historical evolution of psychiatry has to carry this discipline to the research of its diagnostic elements or criteria, more sure and characteristic, and these are constituted by the alterations, anomalies, changes and deficiency of the mental functions. He said that as we had received from Griesinger the first impulse to the neurological study, so we had to receive from Morel and Maudsley the impulse to the study of experimental psychology. He demonstrated how different the present psychology is from the old, and that it possesses the methods, intents and characters of a positive and experimental science, as all others have. The charge that psychiatry makes too much philosophy because it treats psychopathic phenomena, is founded upon the error of still confounding the old with the new psychology, and of considering every psychological research as a metaphysical play. Truly, in the future, the aim of psychiatry must be to harmonize the two tendencies, the somatic and the psychological. Professor Morselli cannot accept the other charge that psychiatry is out of the way, and detaches itself from medical teachings because it has not found the equation between brain and thought. The lecturer mentioned the imperfections of neurology, its errors, and its uncertainties about the structure and functions of the nervous system, for which psychiatry is not responsible.

The idea that every mental disturbance has an organic fundament is correct, but not in the sense given by the anatomo-pathologists of the classic histological school. We cannot suppose that delirium, fixed ideas, and the disorders of sentiment have as equivalents the great alterations of the nervous centers accessible to our means of research. We shall have to resort to micro-chemistry, to micro-physics, and to molecular micro-mechanics. The teachings of

Brown-Sequard on inhibition and dynamogeny can throw much light on this field.

The attempts to join psychiatry with neurology have not given good results. The lecturer showed the faults and imaginations of Luys' scheme, and the imperfections of Meynert's—both of an anatomical kind. He also showed the novelty and the temerity of Arndt's scheme of a physiological basis.

The claim that psychiatry has not given any benefits, is an error and an almost offensive forgetfulness. We owe the doctrine of the anthropological foundation of insanity to psychiatry, especially the Italian, to Lombroso and his school. The lecturer mentioned at length the progress of psychiatry in the last fifteen years, such as the teachings of morbid heredity and its consequences; the degeneration applied to congenital forms of insanity, and the genesis and nature of delirium. He mentioned the studies on insanities or constitutional anomalies of the mind in the relation between psychosis and neurosis; between epilepsy, moral insanity and delinquency; on the influence of biological periods of life and of critical epochs; and on the modifying action of the historical ambient. He also cited the researches on the fundamental lesions of the sentiment; on perceptions, and on the psychometry; on the relation of suicide and insanity; on the pathogenesis of pellagra; on that of progressive paralysis and on the distinction of typical paralytic forms of pseudo-paralysis. He spoke of the applications of these studies to moral science, demonstrating how psychiatry is the natural tie between the medical and social sciences. He closed by describing psychiatry as a curative art, and the enormous progress of the last twenty years in the technique of insane asylums.

On the afternoon of September 20th, Professor E. Maragliano, of Genoa, gave his lecture on "The Climatic Treatment of Phthisis." He showed that purity of air is the most important factor in the treatment of phthisis. The best climate is that of the mountain, for its pure air, low pressure and low temperature. But if the disease has a rapid course, the mountain is to be avoided.

Professor E. Bottini, of Pavia, delivered an important lecture on "The Treatment of Ischuria from Enlarged Prostate Glands." He spoke at length on the disease, and especially on all the instruments and operations for its cure. He exhibited a special instrument, invented by himself, which had acted so far admirably in his hands. König and Thompson are using it with success. It is a thermo-galvanic cauterizer. It consists of a sound having four chambers, in two of which runs the electricity, while in the other two a current of cold water goes and returns. In this manner the whole sound is kept cool, and the galvano-cautery acts only at the exit. The operation gives very little pain. The instrument is easily applied, and the results have always been good.

The instrument was generally approved and praised by all present.

Other lectures were given, as "On Professional Secrecy," by Professor Guelfi, and on "Small-pox and Vaccination in Turkey," by Dr. Violi.

EXPOSITION.

The exposition of surgical instruments and apparatus pertaining to medicine was held at Ghislieri College. It was a well-arranged and fine show. Especially interesting was the collection of special furniture for maniacs and beds for epileptics from the insane asylum of Turin. The war and marine departments exhibited much sanitary material for the time of war. The Italian Red Cross had a beautiful collection of saving, dressing material, models, statistics, etc. Much enthusiasm was aroused by the fine exhibit of the Medical and Surgical Poli-ambulance of Milan. This humanitarian and scientific institution, in only five years, has shown statistics of over eighty thousand patients. The clinical material is accurately collected in registers and nosographical tables; the result of surgical operations in plaster of paris models and photos. Splendid was the show of surgical instruments, antiseptic dressings, electric batteries, etc., by Italian makers. Mosso's phletismograph and sphygmomanometer received much attention. I am delighted to say that Italy has really made gigantic progress in medical mechanics, equal to any other country.

AMUSEMENTS.

The members of the association were pleasantly entertained. The city had a gala appearance. Every building was appropriately decorated with flags, etc. Bands of music and choruses of voices played and sang every evening in all public squares. On September 23rd we enjoyed a fine regatta race. In the evening the river was beautifully illuminated, and there was a splendid show of fireworks on the water. Several receptions were given by the prominent citizens. That given by the prefect and mayor was the most elegant.

CHARITIES.

The sick and the poor were not forgotten. Two collections were made in every section, which netted nice sums of money. One was fo the relief of the sufferers from cholera in southern Italy, and the other for the maintenance and education of orphan children of poor physicians.

The meeting was brought to a close on Saturday, September 24th, with appropriate speeches made by the President, Professor Golzi, Professors Bizzozero, Fedeli, the mayor of Pavia, and others. The meeting was by all pronounced a great success. About eight hundred members were present and the amount of work done was really extraordinary. The meeting lasted five days, and the whole time was one long, uninterrupted period of work. Every section completed its programme. All the local celebrities of medical science joined in the discussions, and helped to clear many important and recent discoveries.

Let, then, the epithet of "Dolce far Niente" no longer be applied to the Italians. They are fast rising to that level upon which they once stood, and were venerated by the whole civilized world.

Padua was selected for the next place of meeting, in 1889.

A. LAGORIO.

Chiavari, Italy.

